# A regulatory role for the co-chaperone FKBP51s in PD-L1 expression in glioma

**DOI:** 10.18632/oncotarget.19309

**Published:** 2017-07-17

**Authors:** Paolo D’Arrigo, Michele Russo, Anna Rea, Martina Tufano, Elia Guadagno, Maria Laura Del Basso De Caro, Roberto Pacelli, Felix Hausch, Stefania Staibano, Gennaro Ilardi, Silvia Parisi, Maria Fiammetta Romano, Simona Romano

**Affiliations:** ^1^ Department of Molecular Medicine and Medical Biotechnologies, Federico II University, Naples, Italy; ^2^ Department of Advanced Biomedical Sciences, Federico II University, Naples, Italy; ^3^ Technical University Darmstadt Institute of Organic Chemistry and Biochemistry, Darmstadt, Germany

**Keywords:** PD-L1, glioblastoma multiforme, FKBP51, ionizing radiation, SAFit

## Abstract

**Background:**

FKBP51 is a co-chaperone with isomerase activity, abundantly expressed in glioma. We previously identified a spliced isoform (FKBP51s) and highlighted a role for this protein in the upregulation of Programmed Death Ligand 1 (PD-L1) expression in melanoma. Because gliomas can express PD-L1 causing a defective host anti-tumoral immunity, we investigated whether FKBP51s was expressed in glioma and played a role in PD-L1 regulation in this tumour.

**Methods:**

We used D54 and U251 glioblastoma cell lines that constitutively expressed PD-L1. FKBP51s was measured by immunoblot, flow cytometry and microscopy. In patient tumours, IHC and qPCR were used to measure protein and mRNA levels respectively. FKBP51s depletion was achieved by siRNAs, and its enzymatic function was inhibited using selective inhibitors (SAFit). We investigated protein maturation using N-glycosidase and cell fractionation approaches.

**Results:**

FKBP51s was expressed at high levels in glioma cells. Glycosylated-PD-L1 was increased and reduced by FKBP51s overexpression or silencing, respectively. Naïve PD-L1 was found in the endoplasmic reticulum (ER) of glioma cells complexed with FKBP51s, whereas the glycosylated form was measured in the Golgi apparatus. SAFit reduced PD-L1 levels (constitutively expressed and ionizing radiation-induced). SAFit reduced cell death of PBMC co-cultured with glioma.

**Conclusions:**

Here we addressed the mechanism of post-translational regulation of PD-L1 protein in glioma. FKBP51s upregulated PD-L1 expression on the plasma membrane by catalysing the protein folding required for subsequent glycosylation. Inhibition of FKBP51s isomerase activity by SAFit decreased PD-L1 levels. These findings suggest that FKBP51s is a potential target of immunomodulatory strategies for glioblastoma treatment.

## INTRODUCTION

Glioblastoma is the most dangerous and aggressive form of brain cancer. Currently, the curative attempt is not possible in the majority of glioblastoma patients. However, there are some long-term survivors. The use of targeting molecules that neutralize the so-called “checkpoint inhibitors” of the immune system is a promising strategy for glioblastoma treatment [1]. Similarly to many other tumours, gliomas express Programmed Cell Death-Ligand 1 (PD-L1) [2–4], encoded by the *PDCD1L1* gene. Although PD-L1 is not an oncogenic driver of the tumour per se, it nevertheless protects the tumour from its immune microenvironment. Engagement of PD-L1, with its cognate receptor PD1, expressed by activated lymphocytes, inhibits T-cell activation via phosphatase SHP2-mediated inhibition of kinases [5]. PD-L1 can also exert an inhibitory effect on T cells through B7-1 [6]. Monoclonal antibodies targeting PD-1/PD-L1 are being extensively used in a wide range of solid tumour types, including melanoma, non-small cell lung cancer, squamous cell cancer of head and neck, bladder cancer, renal cell carcinoma, as well as haematological malignancies, particularly, aggressive B-cell lymphomas and multiple myeloma [7]. The predictive value of PD-L1 as a biomarker of response is under continuous investigation, especially because of the numerous technical issues associated with IHC. The use of various PD-L1 antibodies and diverse IHC cut-offs across clinical trials has made it difficult to assess the usefulness of PD-L1 as a biomarker of response. However, some available data suggest that patients with higher levels of PD-L1 expression have an improved response rate to immunotherapy and progression-free survival [8]. PD-L1 expression in glioblastoma tumours is considerably higher than that observed in melanoma and lung cancer [3]. Also, PD-L1 expression in glioma is sustained and increased by standard radiation treatments [9, 10]. Based on their encouraging efficacy in an increasing variety of human neoplasias, several clinical studies are currently under way to evaluate checkpoint inhibitors in patients with glioblastoma [11–13]. A major concern of the immune checkpoint blockade remains immunotoxicity, with related adverse events that are sometimes life-threatening and prevent the completion of the treatment. In addition, the occurrence of primary or acquired resistance to the inhibitory immune checkpoint-targeted therapy represents a further limitation of this anti-cancer approach. A better understanding of the specific mechanisms that regulate PD-L1 expression may provide new tools for circumventing the tumour’s ability to suppress its immune microenvironment. Parsa et al. demonstrated the dependence of PD-L1 expression on the PI3k/Akt pathway in human glioma and showed a post-transcriptional increase of such immune-inhibitory ligands after the loss of PTEN [14]. Similarly to several other membrane proteins, PD-L1 expression is also regulated at a post-translational level. PD-L1 undergoes heavy glycosylation that greatly increases the affinity for PD1 [15]. The targeting of mechanisms that control post-translational modification can help in neutralizing the pro-tumoral effect of PD-L1. Our research has previously identified a role for a spliced variant of the FKBP5 gene in the regulation of PD-L1 expression in melanoma [16]. FKBP5 encodes a large immunophilin which was originally cloned in lymphocytes [17] but also found abundantly expressed in several tumours [18–23], including glioblastoma [24]. The FKBP51 protein structure includes a C-terminal TPR three tandem repeat domain, responsible for protein-protein interaction, and two N-terminal FK506 binding domains (FKBP), of which the most N-terminal one exerts a peptidyl-prolyl isomerase (PPIase) activity [25]. The spliced isoform (isoform 2 or FKBP51s) [16] lacks a TPR domain and has a distinct C-terminus compared to isoform 1. Isoform 2 resulted induced, bidirectionally, in melanoma and lymphocytes following their interaction through the PD-L1/PD1 ligand/receptor system [16]. Classically, the PPIase activity of FKBPs is inhibited by the immunosuppressant agents rapamycin and FK506. More recently, potent and specific inhibitors of FKBP51 have been created and named SAFit 1 and 2 because of their selectivity through an “induced fit” mechanism [26]. Here we aimed to investigate the role of FKBP51s in PD-L1 regulation in glioma. FKBP51s showed varying degrees of expression in glioma cancer cell lines and tumours from patients. Silencing of FKBP51s reduced the expression levels of glycosylated PD-L1 forms. FKBP51s physically interacted with the naïve PD-L1 protein in the endoplasmic reticulum (ER), suggesting it served as a catalyst of PD-L1 glycosylation. We also show that SAFit1 and, especially, SAFit2 reduced expression of PD-L1 and impaired ionizing radiation (IR)-induced PD-L1 up-regulation.

## RESULTS

### Expression of FKBP51s in glioma cells

**Figure 1:**
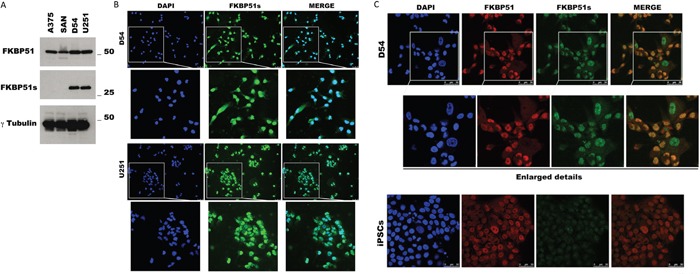
FKBP51s expression in glioma cells **(A)** Immunoblot assay of FKBP51 and FKBP51s expression in D54 and U251 glioma cell lines. Lysates from A375 and SAN melanoma cell lines were run for comparison. γ-Tubulin was used as loading control. **(B)** FKBP51s (green) immunostaining of D54 and U251. DAPI (blue) was used to counterstain nuclei; FKBP51s/DAPI merge was shown. Scale bar 50μm. Image was captured with an inverted microscope (DMI4000; Leica Microsystems, Heidelberg, Germany) and Leica Application Suite Advanced Fluorescence (LAS AF) software (Leica Microsystems). **(C)** D54 glioma cell and the undifferentiated human iPSC were stained with DAPI (blue) to counterstain nuclei; FKBP51 (red); FKBP51s (green); FKBP51/FKBP51s merge was shown. Scale bar 50μm. Confocal images were captured with Leica TCSSMD FLIM (Leica Microsystems) microscopes.

We measured the expression of FKBP51 isoforms by immunoblot in D54 and U251 glioma cells, using A375 and SAN melanoma cell lines for comparison. As shown in Figure 1A, both the canonical and the spliced FKBP51 isoforms were expressed in the glioblastoma cells. Whereas in melanoma, FKBP51s was barely detected under basal conditions [16]. FKBP51s expression in glioblastoma cells was confirmed by fluorescent microscopy (Figure 1B), showing a localization in both nuclear and cytoplasmic compartments (Figure 1B). A co-staining of both isoforms showed that the canonical and the spliced FKBP51 are coexpressed, even if the canonical isoform seems to have a brighter nuclear signal than the short isoform (Figure 1C). The co-staining also suggested that, in some glioma cells, the short isoform appears as the only detectable form in the nucleus. Figure 1C also suggests the specificity of the FKBP51s signal, as it was barely detectable in another cell line (iPSCs, undifferentiated human induced Pluripotent Stem Cell) that was found to express the canonical isoform.

### FKBP51s regulates PD-L1 expression

**Figure 2:**
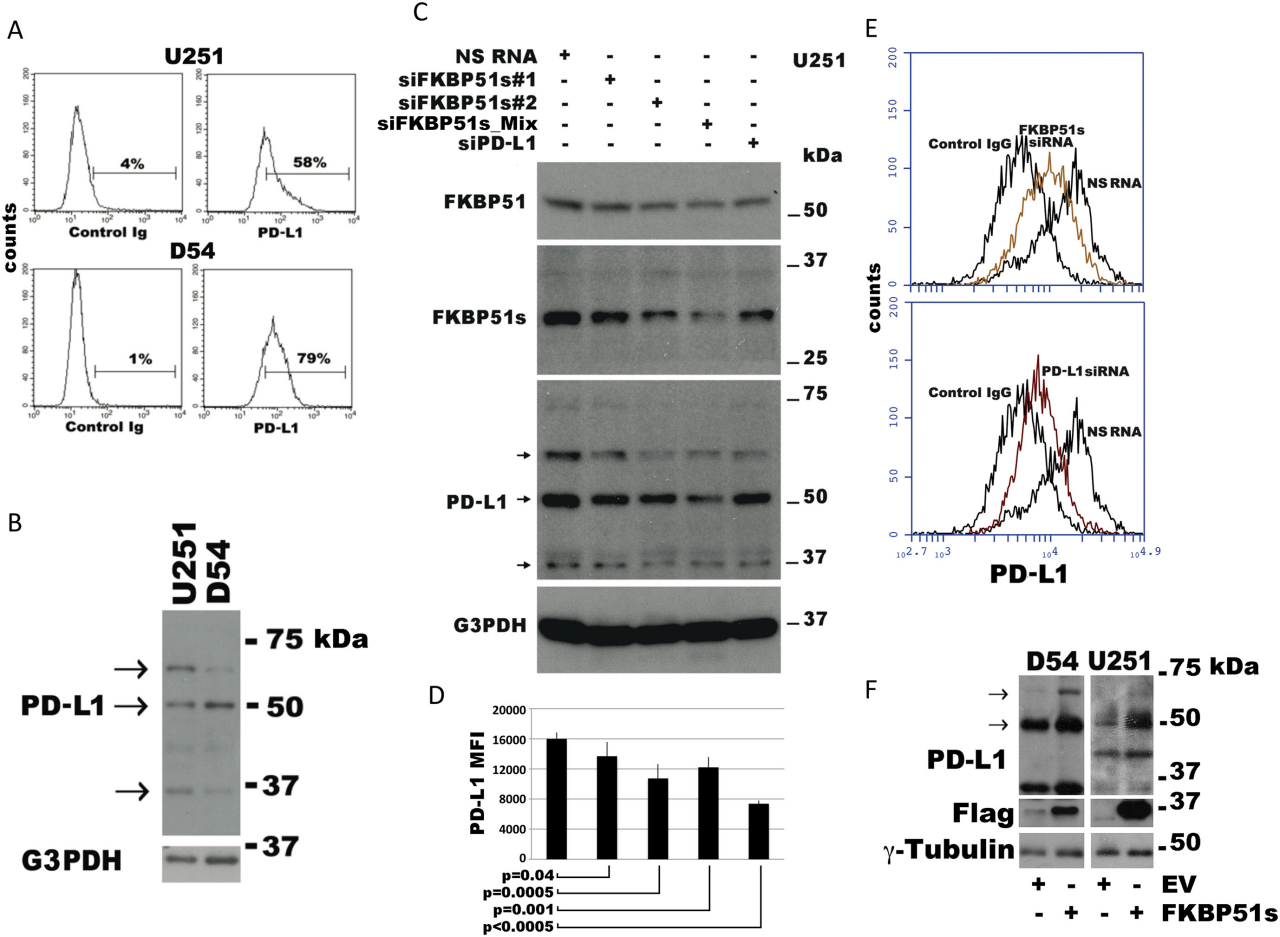
Modulation of PD-L1 expression in glioblastoma cells **(A)** Flow cytometric histograms of PD-L1 expression in U251 and D54 glioblastoma cells. **(B)** Immunoblot of lysates prepared from the same cells shows 3 isoforms ascribable to the naïve (37 kDa) and post-translational modified protein (∼50 and ∼68 kDa). **(C)** Immunoblot of cell lysates obtained from the glioblastoma cell line U251, transfected with FKBP51s siRNAs, PD-L1 siRNA or a non-silencing (NS) RNA. G3PDH was used as loading control. **(D)** U251 cells treated as in C, was analysed by flow cytometry for PD-L1 expression. The graph represents MFI (mean and standard deviation) of PD-L1 expression. P values are indicated (N=6). **(E)** Flow cytometric histograms of PD-L1 expression in U251 glioma cells, silenced or not for FKBP51s, or PD-L1. Histograms are shown in overlay. **(F)** Immunoblot of cell lysates obtained from the D54 and U251 glioblastoma cell lines over expressing FKBP51s or transfected with the void vector (EV). Expression of PD-L1 resulted increased by FKBP51s but not EV transfection. γ-Tubulin was used as loading control.

To investigate the role of FKBP51s on PD-L1 expression regulation in glioma, we first measured PD-L1 levels in U251 and D54 glioblastoma cell lines by flow cytometry (Figure 2A) and immunoblot (Figure 2B). Both cell lines expressed high levels of PD-L1 on the plasma membrane (Figure 2A). Several PD-L1 isoforms were observed by immunoblot (Figure 2B). A lower band (at ∼37 kDa) corresponding to the naïve protein and two upper bands at ∼50 and ∼68 kDa could be accounted for by post-translationally modified isoforms [15, 16]. However, as other authors and we noted [32], PD-L1 expression has a highly variable pattern and banding, even using the same antibody for detection. Such variability is mostly because of post-translationally modified forms or different degrees of protein maturation [32]. Consistently, in our study, additional bands between 37–50 kDa or under 37 kDa were sometimes detected. Next, we used different FKBP51s siRNAs and a PD-L1 siRNA for protein knockdown and evaluated modulation of PD-L1 levels by immunoblot. Figure 2C shows that both FKBP51s siRNAs #1 and #2 (but especially the siRNA #2 and the siRNA combination) were effective at reducing PD-L1 levels in U251 glioma cells. The intensity of the 68-kDa band was reduced with both FKBP51s siRNA and PD-L1 siRNA. The 50-kDa band was reduced when cells were treated with FKBP51s siRNAs and especially the mix of siRNAs, which strongly reduced FKBP51s levels. A decrease of the 37 kDa PD-L1 band was observed with PD-L1 siRNA and, surprisingly, also under FKBP51s silencing. The level of the PD-L1 transcript of U251 and D54 cells was reduced by PD-L1 siRNA but not FKBP51s silencing (under the latter condition, PD-L1 transcript appeared to be slightly increased) (Supplementary Figure 1). Apparently, this result excluded a transcriptional regulation by FKBP51s and, thus, the decrease in the band at 37 kDa could reflect a condition of ER stress related to possible imbalance of ER-proteins. The observation that intensity of the highest kDa bands was reduced by FKBP51s siRNA more than PD-L1 siRNA was consistent with our hypothesis that FKBP51s is involved in PD-L1 post-translational control. The effect of FKBP51s knockdown on PD-L1 was also evaluated by flow cytometry. Analysis of mean fluorescence intensities (MFI) of PD-L1 expression on the plasma membrane of U251 cells suggested a significant decrease in PD-L1 expression following FKBP51s silencing (Figure 2D). Representative PD-L1 histograms in the overlay, from U251 cells, are shown in Figure 2E. Finally, to confirm that FKBP51s positively regulated PD-L1 expression in glioma, we overexpressed this FKBP51 isoform in D54 and U251 cells and measured PD-L1 level by immunoblotting. Figure 2F shows that the ∼50 and 68 kDa PD-L1 bands were increased by FKBP51s-overexpression compared to control cells transfected with empty vector. Supplementary Figure 1B shows the mRNA levels of PD-L1 and FKBP51s in transfected D54 and U251 cells.

### FKBP51s serves as a PD-L1 co-chaperone in ER

**Figure 3:**
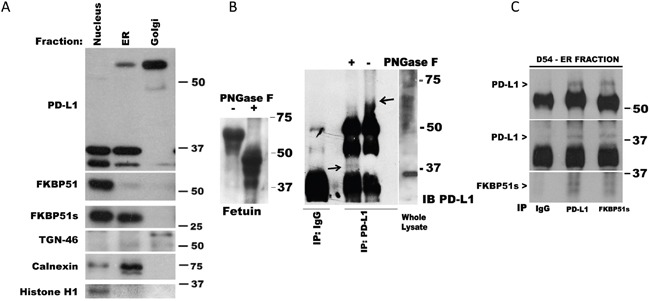
FKBP51s is associated with PD-L1 in ER **(A)** Immunoblot of D54 lysates obtained from sub-cellular compartments. PD-L1, FKBP51 and FKBP51s levels are shown along with relative organelle markers. **(B)** Whole D54 lysates immunoprecipitated with anti-PD-L1 and subjected, or not, to PNGase F treatment; the arrows indicate the higher (− PNGase F) and lower (+ PNGase F) PD-L1 band. Fetuin, on the left of the panel, was used as positive control of PNGase digestion. **(C)** Co-IP of PD-L1 and FKBP51s in ER fraction. ER lysate was immunoprecipitated with anti-PD-L1 and anti-FKBP51s and recognized for each protein by immunoblot.

The addition of carbohydrates is the principal chemical modification to most plasma membrane proteins. PD-L1 is N-glycosylated (http://www.uniprot.org/uniprot/Q9NZQ7#ptm_processing). Because glycosylation can occur in the lumen of the ER and/or in the lumina of the Golgi cisternae, to address the role of FKBP51s in PD-L1 glycosylation, we performed subcellular fractionation of D54 cells and obtained protein extracts from these cell compartments. As shown in Figure 3A, the naïve 37 kDa PD-L1 was detectable in the ER, and scarcely in the Golgi. The 68-kDa band was expressed mostly in the Golgi. In the Golgi, the band at 50 kDa was also appreciable. FKBP51s, but not FKBP51, was found in the ER. The naïve PD-L1, FKBP51 and FKBP51s were all found in the nucleus. By using an N-glycosidase, we could confirm that the 68-kDa band was glycosylated. As shown in Figure 3B (right), treatment of the immunoprecipitated PD-L1 protein with PNGase F produced a decrease of the 68-kDa band (indicated by the right arrow) and the appearance of an additional band at around 37 kDa (indicated by the left arrow). These findings suggest that FKBP51s has a role in catalysing PD-L1 folding, an essential step of glycosylation. To address this issue, we performed co-IP of protein from purified ER extract. As shown in Figure 3C, pull-down of either FKBP51s or PD-L1 confirmed that the two proteins interact with each other in the ER.

### SAFits, the selective inhibitors of FKBP51, reduce PD-L1 expression

**Figure 4:**
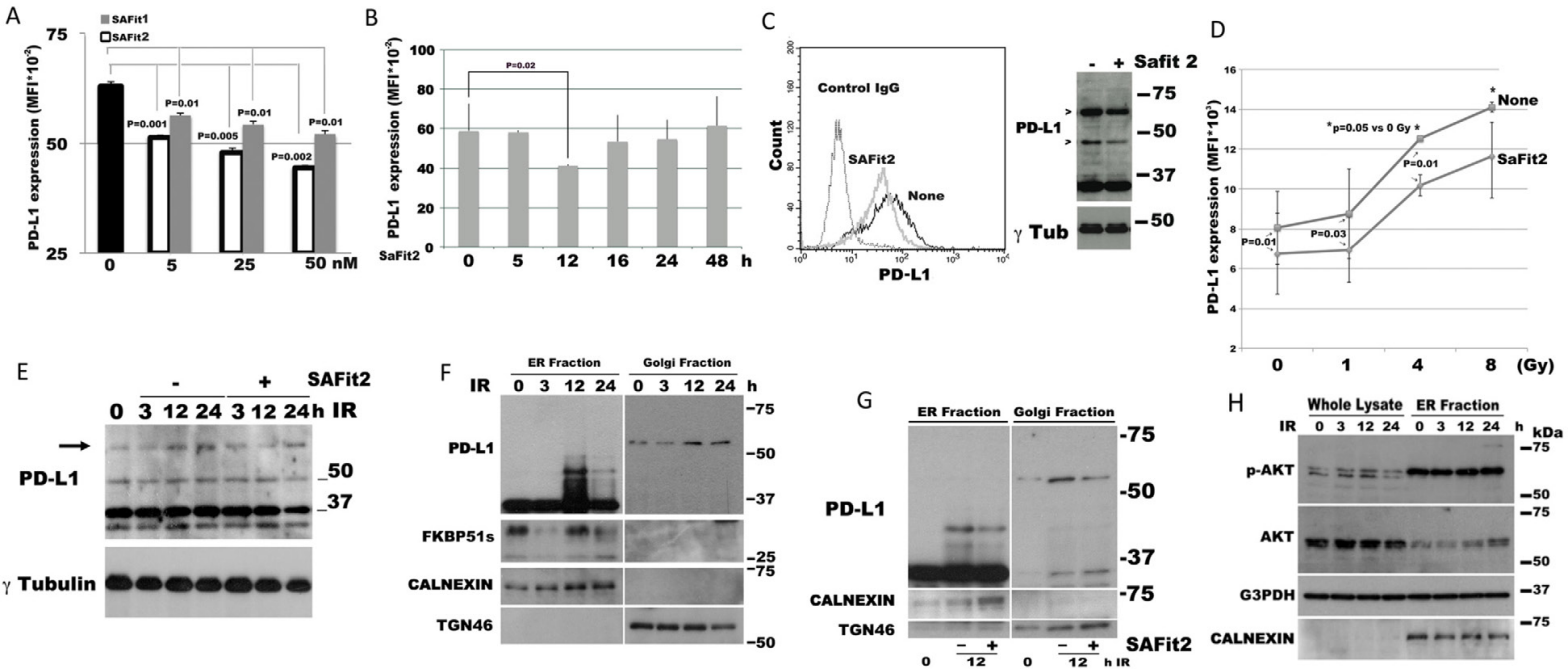
SAFit reduces the expression of PD-L1 **(A)** Dose response assay of SAFit on PD-L1 expression. D54 glioblastoma cells were incubated with SAFit1 and 2 at different concentrations. After 12 h of incubation, cells were harvested and analysed by flow cytometry for PD-L1 expression. Graph columns represent mean fluorescence intensities and bars the standard deviation of PD-L1 expression (N=3). **(B)** Kinetic of PD-L1 expression. Glioblastoma cells were incubated with SAFit2 at different times, then, cells were harvested and analysed by flow cytometry for PD-L1 expression. Graph represents mean fluorescence intensities of PD-L1 expression (N=3). **(C)** Flow cytometry (left) and immunoblot (right) of SAFit-modulated PD-L1 expression. Cells were cultured, in the absence or the presence of 50 nM SAFit2 for 12h. **(D)** Dose response assay of IR-induced PD-L1 expression and modulation by SAFit2. D54 glioblastoma cells were irradiated at different Gy doses (1, 4 and 8Gy), in the absence or the presence of 50 nM SAFit2. After 12 h incubation, cells were harvested and analysed by flow cytometry for PD-L1 expression. The graph represents mean fluorescence intensities (mean and standard deviation, N=3) of PD-L1 expression. **(E)** Kinetic of IR-induced PD-L1 expression levels. D54 cells were harvested 3, 12 and 24h after 4 Gy IR. The band at 68 kDa (indicated by the arrow) resulted increased after 12 and 24 h from IR. The addition of SAFit prevented such an increase. **(F)** Immunoblot of lysates obtained from sub-cellular compartments of D54 cells after a 4 Gy IR. PD-L1 and FKBP51s levels are shown along with relative organelle markers. **(G)** Immunoblot of D54 lysates obtained from sub-cellular compartments. D54 cells cultured in the presence or not of SAFit2, was harvested 12h after IR. PD-L1 is shown along with relative organelle markers. SAFit2 reduced ER and Golgi PD-L1 bands. **(H)** pAkt expression levels in D54 lysates obtained from whole cell and ER compartment, shown along with ER marker.

Because SAFit compounds selectively inhibit the catalytic activity of FKBP51, we investigated their ability to modulate expression of PD-L1 in glioma cells. Figure 4A shows a dose response assay of PD-L1 expression levels, measured by flow cytometry, after 12 h of culture in the presence of one of three doses of SAFit1 and SAFit2 (5, 25 or 50 nM). Both compounds (but especially SAFit2) produced a decrease in PD-L1 expression on the plasma membrane, as measured by flow cytometry. SAFit2 significantly inhibited PD-L1 activity at 12 h, but activity returned to the baseline after 48 h (Figure 4B). Figure 4C shows a representative flow cytometry histogram of PD-L1 down modulation by SAFit2 (left) and an immunoblot (right) showing that the post-translational modified forms of PD-L1 (indicated by the arrows) are especially affected by SAFit2. Because ionizing radiation *increases tumour* cell expression of *PD-L1* [10], we investigated whether SAFit was able to counteract such an IR effect. Using different IR doses, we detected a significant increase in PD-L1 expression at 4 and 8 Gy (p=0.05), compared with non-irradiated glioma cells (Figure 4D). Upregulation of PD-L1 after IR was also measured at the mRNA level (Supplementary Figure 2). SAFit2 produced a decrease in PD-L1 expression. In the presence of SAFit2, PD-L1 expression following 4 Gy irradiation was similar to the untreated control cells (Figure 4D). An immunoblot of D54 cell extracts prepared at 0, 3, 12 and 24 h following irradiation showed an increase of the intensity of the band at 68 kDa (indicated by the arrow) after 12 and 24 h (Figure 4E). Such an increase appeared to be counteracted by SAFit2 (Figure 4E). The blot also shows two bands of around 37 kDa. The lower of these bands was especially induced at 12 h after irradiation. This approximately 34-kDa band might correspond to a cleavage product or further isoform of PD-L1, which is increased by IR. The blot also shows a decrease of the 37 kDa band after 24 h of SAFit plus IR, which suggests that SAFit might affect protein synthesis. Analysis of extracts obtained by cell fractionation (Figure 4F) shows that, in the ER, IR induces the expression of PD-L1 forms of between 37 and 50 kDa (at 12 h after irradiation) but that these are decreased at 24 h. Interestingly, FKBP51s disappeared from the ER at 3 h after IR, reappearing at 12 h after IR. Golgi extracts showed that the levels of the PD-L1 upper band changed in accordance with the upper band of whole extracts. To address the effect of SAFit on IR-induced PD-L1 upregulation, we irradiated the cells in the absence or the presence of 50 nM SAFit2 and recovered extracts after 12 h. By this approach, we confirmed that IR induces the expression of PD-L1 forms of up to 37 kDa and that this effect is attenuated by SAFit2 (Figure 4G). The role of FKBP51s in IR-induced PD-L1 increase was further investigated using a siRNA for FKBP51s in irradiated cells. As shown in Supplementary Figure 3, FKBP51s silencing impaired IR-induced PD-L1 upregulation.

In addition, we thought to investigate the levels of pAkt during IR, given that Akt is the master regulator of PD-L1 expression in glioma [11]. Phospho-Akt levels were increased at 3 h after IR, remained high at 12 h after IR, and decreased at 24 h after IR (Figure 4H). An analysis of pAkt levels in ER extracts found that these levels were particularly high in this cell compartment (Figure 4H), which is consistent with the role of Akt in the ER membrane [33]. The observation that the PD-L1 maturation forms appeared in the ER only upon restoration of the FKBP51s level, is a further element in support of the important FKBP51s function as a PD-L1 molecular chaperone in ER.

### SAFits reduce PD-L1-induced cell death of PBMC cocultured with glioma cells

**Figure 5:**
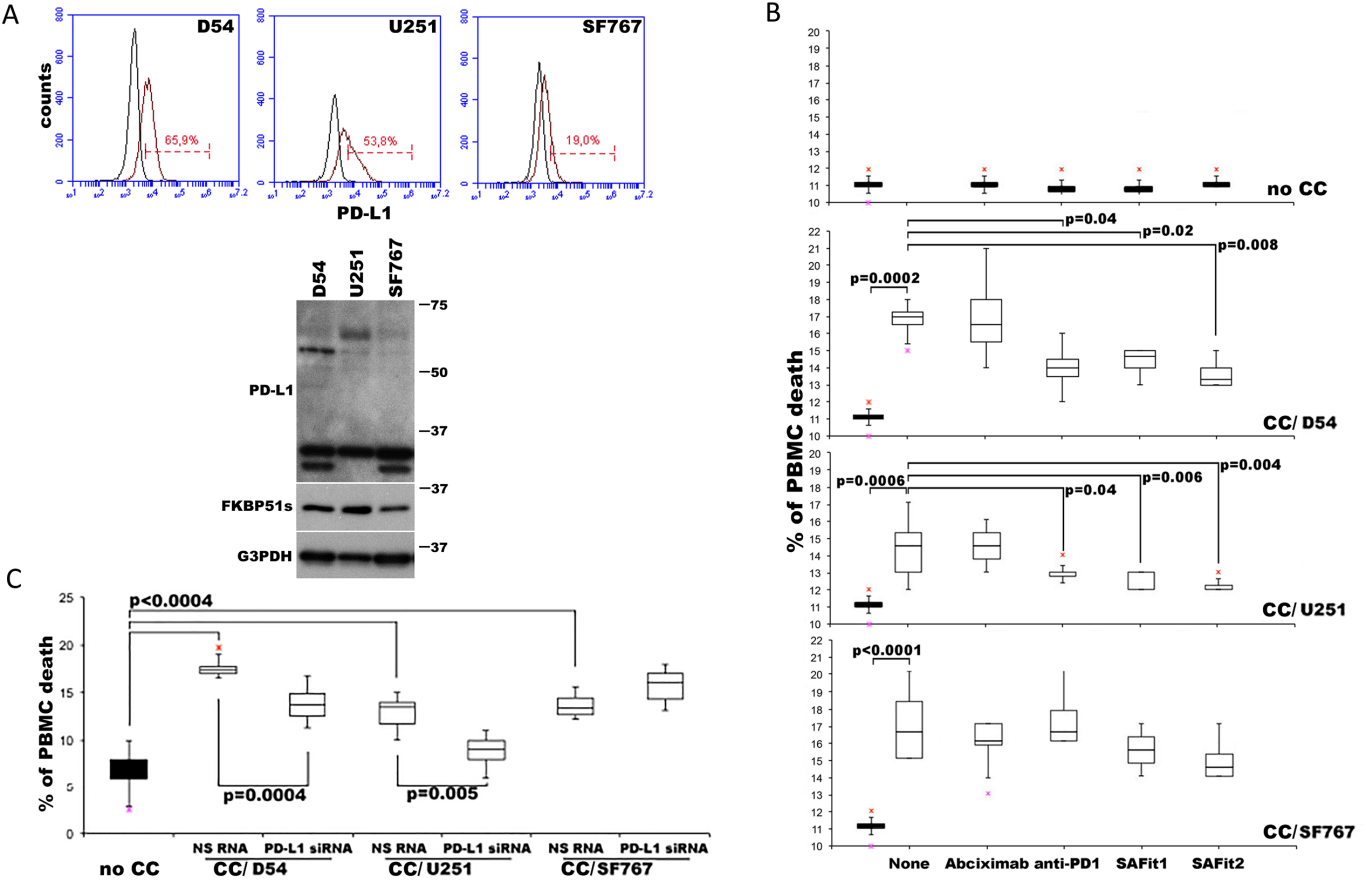
PD-L1-induced PBMC death is reduced by SAFit **(A)** PD-L1 expression in D54, U251 and SF767 glioblastoma cells analysed by flow cytometry (upper) and immunoblot (lower). **(B)** Graphical representation of cell death values of PBMC not cocultured (no CC) or cocultured (CC/) for 6 h with D54, U251 and SF767 glioblastoma cells, in the absence or presence of anti-PD1, SAFit1, SAFit2 or a humanized antibody (abciximab) as control. PBMC was harvested and assayed in flow cytometry for Annexin-V binding in double fluorescence with CD45. Values measured for each cell line are reported as box plots. Differences (p-values) are shown (N=6). **(C)** Graphical representation of cell death values of PBMC no CC or cocultured for 6 h with D54, U251 and SF767 glioblastoma cells, previously silenced or not for PD-L1. PBMC was harvested and assayed by flow cytometry. Values are reported as box plots. Differences are shown (N=6).

The PD1 receptor was identified as a gene upregulated in a T-cell hybridoma undergoing cell death [34]. Accordingly, triggering PD1 through PD-L1 induces cell death [35]. We performed co-cultures of glioblastoma and healthy donor PBMCs, as previously described [16], to address whether SAFit-mediated PD-L1 down modulation on glioma cells could modulate death of PBMC via tumour-associated PD-L1. To this end, we used different glioma cell lines, D54, U251 and SF767, the latter of which expresses very low levels of PD-L1 (Figure 5A) and performed PBMC/glioma co-cultures. PBMCs were harvested after 6 h and analysed by annexin V staining in double fluorescence with CD45, by flow cytometry. A gate was placed on CD45 positive cells to exclude non-PBMC cells from the analysis (Supplementary Figure 4). Figure 5B shows box plots of cell death values of PBMC not co-cultured (no CC) or co-cultured with glioma cells (CC). We measured a significant increase in the cell death of PBMCs co-cultured with glioma cells, compared to cell death of no CC PBMCs. The addition of anti-PD1, but not an unrelated humanized antibody (abciximab) [27], reduced cell death in D54 and U251 but not in SF767 co-cultures (Figure 5B). These results suggest that glioma cells can kill PBMCs through PD-L1. However, the observation that CC/SF767 PBMC also undergoes cell death suggests a tumoral-induced cytotoxicity independent of PD-L1, possibly involving other factors (e.g. Fas ligand [36] or TGF-β [37]). A further detailed analysis for comparison of apoptosis of each experimental condition is shown in Supplementary Table 1. Use of SAFit1 and SAFit2 also resulted in protection of CC/D54- and CC/U251-PBMC from cell death, in accordance with the PD-L1 down modulation effect by SAFit. Additionally, the use of PD-L1 siRNA, to down modulate PD-L1 on glioma cell lines, reduced CD45^+^ cell death in D54- and U251- but not SF767- co-cultures (Figure 5C), thus supporting the causative role of PD-L1 in glioma killing of immune cells.

### FKBP51s is expressed in glioblastoma specimens from patients

**Figure 6:**
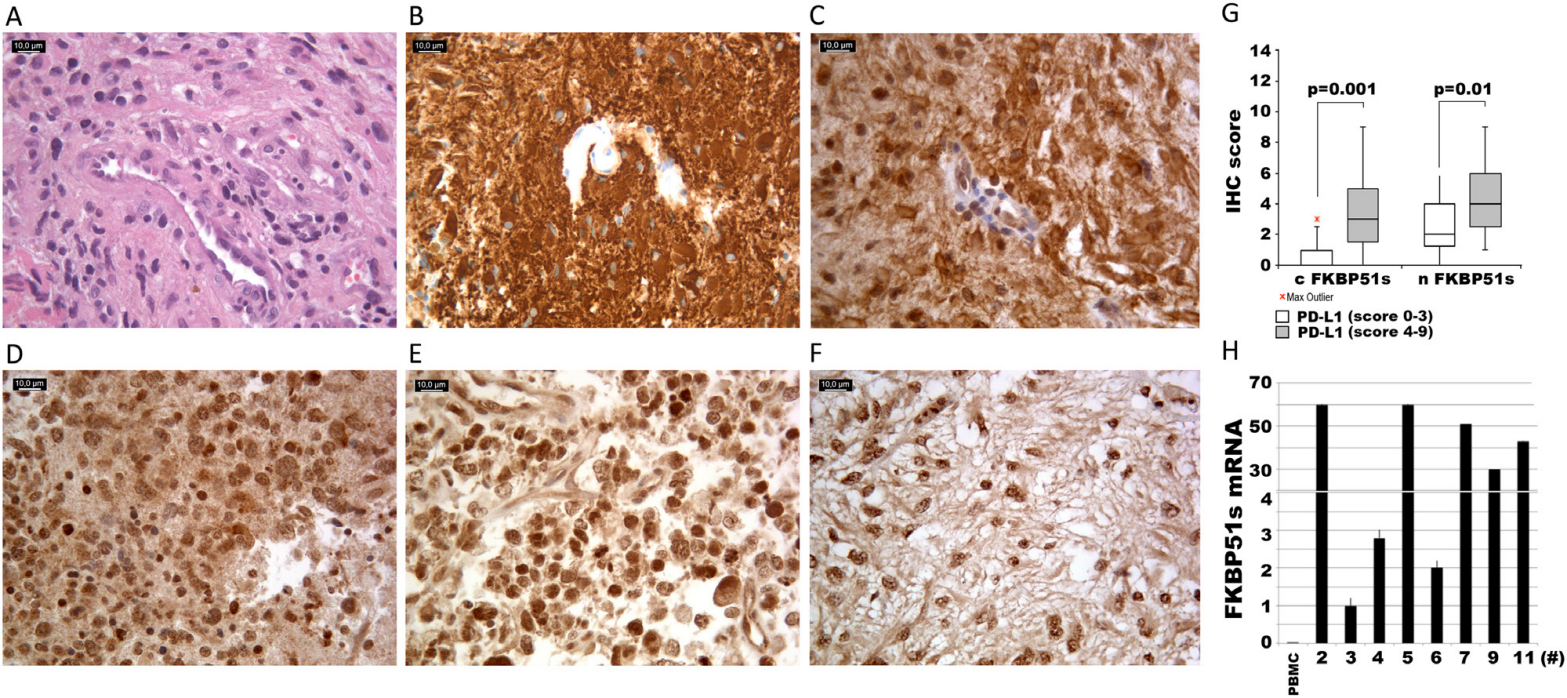
FKBP51s and PD-L1 immunohistochemistry of glioblastoma samples Case #28, **(A)** hematoxylin and eosin staining; **(B)** anti-GFAP; **(C)** diffuse/fibrillary cytoplasmic PDL1 expression with a membranous signal in more than 5% of neoplastic cells. Original magnification x400. **(D, E, F)** FKBP51 immunostaining: cytoplasmic signal was strong, moderate and mild in case #12 **(D)**, #14 **(E)** and #19 **(F)**, respectively. In the nucleus, the reactivity was moderate in case #12 **(D)** and case #19 **(F)** and strong in case #14 **(E)**. Original magnification x400. **(G)** box plots of FKBP51s scores (c=cytoplasmic and n=nuclear) in PD-L1^low^ and PD-L1^high^ expressing glioblastomas. **(H)** quantitative FKBP51s expression of 8 glioblastoma samples. QPCR was performed by using a coamplified β-actin internal control for sample normalization. FKBP51s value of a PBMC has been also run for comparison. Columns and bars are representative of means and standard deviations of values for each sample as individual data point.

Expression of FKBP51s and PD-L1 was investigated by immunohistochemistry in 29 glioblastoma specimens. Details on IHC semi quantitative evaluation are reported in Supplementary Table 2. PD-L1 staining was observed in glioblastoma tissues to a variable extent and intensity. In the majority of the cases, 22/29 (75.86%), a prominent cytoplasmic diffuse/fibrillary expression pattern was observed. In 3 of these 22 cases (3/29; 10.34%), we found interspersed singular or focally aggregated epithelioid cells, with membranous immunostaining for PD-L1. Very faint and focal immunoreactivity was observed in perilesional brain tissue, when present. In Figure 6A, and 6C, hematoxylin and eosin staining, expression of the glial fibrillary acidic protein and PD-L1 expression of case #28 are shown. Two further PD-L1 IHC results are shown in Supplementary Figure 5. Cytoplasmic and nuclear FKBP51s expressions of tumour tissue were observed (Figure 6D–6F). Both of these were scored. The semi quantitative distribution of cytoplasmic FKBP51s was as follows: score 0 in 10 of 29 cases (35%), score 1 in 6 of 29 cases (21%), score 2 in 1 of 29 cases (3%), score 3 in 5 of 29 cases (18%), score 4 in 3 of 29 cases (10%), score 6 in 3 of 29 cases (10%) and score 9 in 1 of 29 cases (3%). Nuclear FKBP51s scores were 0 in 2 of 29 cases (7%), score 1 in 3 of 29 cases (10%), score 2 in 8 of 29 cases (28%), score 3 in 1 of 29 cases (3%), score 4 in 6 of 29 cases (21%), score 6 in 6 of 29 cases (21%), score 9 in 3 of 29 cases (10%). Statistical analysis revealed that the scores of FKBP51s were different when comparing specimens with absent/low-PD-L1 (arbitrarily defined as a score >=0, <=3) and high-PD-L1 (score >=4, <=9). As shown in Figure 6G, the levels of FKBP51s, both cytoplasmic and nuclear, are likely to be increased in high-PD-L1 samples, which is in line with *in vitro* findings that FKBP51s drives PD-L1 expression. FKBP51s expression was confirmed at the mRNA level in eight glioblastoma specimens (Figure 6H).

## DISCUSSION

Here, we address the expression and function in glioma of FKBP51s [10], a protein isoform that is generated by an alternative splicing of the FKBP5 gene. FKBP51s was first identified in melanoma [16]. Unlike in melanoma, in which FKBP51s was upregulated by contact with lymphocytes [16], this spliced isoform appeared to be constitutively expressed in glioblastoma cell lines at high levels. Our results show that, in glioma cells, FKBP51s is complexed with PD-L1 in the ER and its down modulation decreased the level glycosylated-PD-L1, suggesting that the isomerase activity of FKBP51s was relevant for PD-L1 folding and processing. It is worth noting that post-translational modifications of surface receptor/ligand antigens greatly influence the affinity for the cognate molecules. In particular, PD-L1 has an affinity for PD1 of 0.77 μM when glycosylated and in dimers, while the monomeric nonglycosylated protein has an affinity of 26.6 μM [15]. In accordance with its proposed role as a PD-L1 foldase, use of selective inhibitors of FKBP51 PPIase activity significantly down modulated PD-L1 expression. Most interestingly, SAFit2 was able to counteract the increase in PD-L1 levels in glioblastoma cells treated with IR. This result might have an impact in clinical practice, considering that combining IR and anti-PD-L1 treatment led to tumour regression and prevented the growth of secondary tumours in a mouse tumour xenograft model [38]. Given the above findings, FKBP51s may contribute to an efficient inhibitory checkpoint signal, and thus to glioma-edited immune suppression. Consistent with such a hypothesis, the addition of SAFit to co-cultures of PBMCs and glioma cells reduced apoptosis of immune cells induced through PD-L1/PD1. Future *in vivo* studies with syngeneic mouse models are now needed to validate the efficacy of selective FKBP51 inhibitors in modulating tumour PD-L1 expression and, most importantly, to verify whether these small molecules are able to positively affect tumour microenvironment and ameliorate immune defences against glioma. Besides the tumour, immune accessory cells in the microenvironment can express PD-L1 and suppress CD8-mediated tumour killing. Particularly, in glioblastomas, a very recent study [39] identified a tumour-infiltrating myeloid cell (TIM) population that expands in response to dendritic cell vaccine treatment and greatly contributes to PD-L1 expression in the glioblastoma environment. Whether or not this TIM expresses FKBP51s is unknown. Interestingly, we identified a myeloid-derived suppressor cell CD14^+^PD-L1^+^FKBP51s^+^ in peripheral blood of melanoma patients, expressing high TGF-β levels and apparently associated with resistance to ipilimumab [40]. Immunohistochemistry of FKBP51s performed on 29 tumoral samples showed an expression pattern (with either a nuclear and cytoplasmic localization) similar to that observed in cancer cell lines, thus reinforcing the translational implication of our findings. Regarding PD-L1 expression, in our series, the majority of cases showed a cytoplasmic pattern of reactivity, while a membranous signal was faintly evident only in a few tumour cells showing epithelioid morphologic features, as it was previously observed [3]. A possible explanation for this diffuse/fibrillary cytoplasmic pattern has been attributed to the membrane-bound PD-L1, within the delicate tumour cell process forming the pathognomic neurofibrillary matrix of gliomas [3]. Interestingly, we found that the scores of FKBP51s tended to be increased in accordance with PD-L1 scores, which generates the hypothesis (to be addressed with a larger sample size) that the two variables (FKBP51s and PD-L1) might be associated. We observed that 6 out of 22 PD-L1 positive tumour samples expressed only nuclear FKBP51s, which is in disagreement with our finding that the PD-L1 maturation occurs in the ER. An active FKBP51s shuttling within cell compartments, to convey information on nuclear and cytoplasmic activities, as it occurs for several proteins [41], might explain this result. Currently, the biological significance for the proposed shuttling, as well as the function of FKBP51s inside the nucleus, remain obscure.

Given the above, we propose that FKBP51s is a novel element that regulates PD-L1 expression in glioma cells. This co-chaperone functions as a catalyst and foldase in PD-L1 post-translational modifications, occurring during protein maturation and expression on the plasma membrane. *In vitro* inhibition of this protein, either by silencing or chemical agent results in PD-L1 down modulation in glioblastoma cells lines and, as a consequence, the suppressive effect exerted by the tumour on immune cells is relieved. Moreover, targeting of FKBP51s counteracts the PD-L1-increasing effect of ionizing radiation. Our findings on patient tumours show that the spliced FKBP51 is widely expressed in glioblastomas. It is worth noting that factors other than FKBP51s control the expression of PD-L1 at the transcriptional and translational level. Nevertheless, this foldase appears to be important for PD-L1 glycosylation, which increases the affinity for its cognate molecule PD1 [15]. It is noticeable that even if targeting the PD1/PD-L1 inhibitory checkpoint is, to date, the most effective immunotherapy used in the treatment of cancers, primary resistance occurs in almost 60% of patients [42]. In addition, a proportion of the responder patients develop acquired resistance. In general, very little is known about the mechanisms of resistance. A number of factors account for primary resistance, such as poor tumour immunogenicity, limited intra-tumoral T-cell infiltration, defective antigen presentation and naive T-cell priming [42]. The release of adenosine or indoleamine 2,3-dioxygenase in the microenvironment or mutations of the genes encoding *JAK1* or *JAK2* (key intracellular signalling mediating tumour sensitivity to interferon) or the gene encoding β-2-microglobulin (involved in folding and transport of MHC class I molecules) are recognized causes of acquired resistance [42]. We do not have sufficient knowledge on the spliced FKBP51 to speculate on potential effects of its inhibitors on circumventing resistance to anti-PD1. Our data support the conclusion that SAFit acts in the same direction of immune checkpoint blockade (i.e., contrasting PD-L1). In this view, used in combination, SAFit can reduce both the doses and side effects of the immunomodulatory treatment. In addition, a previous study on melanoma [30] showed that SAFit efficiently suppresses the pro-oncogenic activity of canonical FKBP51, namely NF-κB transcription factor activation and cyclin D upregulation. Other authors have identified that canonical FKBP51 exerts a relevant role in glioma aggressiveness [24]. Based on these considerations, SAFit could, at the same time, contrast some autonomous tumour cell functions and PD-L1-mediated T-cell impairment, in glioma tumours, thus integrating two important approaches for cancer treatment. Future studies will address this hypothesis. Finally, the low molecular weight of SAFit allows the agent to penetrate through the brain barrier, which is an important requirement for drugs candidate to glioma treatment [26]. In conclusion, our finding provides the basis for a novel molecular player in glioma immune evasion. *In vivo* studies with a syngeneic mouse glioma model are needed to confirm that FKBP51s is a useful target for control of glioma immune evasion, thus offering a new tool in the immunotherapy armamentarium against cancer.

## MATERIALS AND METHODS

### Cell culture and transfection

Human glioma cell lines D54 and U251, from the CEINGE cell bank (Cellular Technology Platform) at the Advanced Biotechnology Institute (Naples, Italy), were cultured at 37°C and 5% CO_2_ in DMEM-F12 media (Biowest, Nuaillé, France) containing 10% foetal bovine serum (FBS; Biowest). In accordance with the international guidelines established by the United Kingdom Coordination Committee on Cancer Research (UKCCCR) for the good use of continued cell lines, the cell bank of CEINGE guarantees cell lines for identity, cross-contamination and microorganism contamination. Pluripotent Stem Cells (iPSC, iPS DF19-9-7T; WiCell, Madison, WI, USA) were grown on Matrigel (BD Biosciences) coated dishes in mTeSR1 medium (Stem Cell Technologies). Human glioma cell line SF767 was kindly provided by Prof. Gerolama Condorelli (University of Naples, Federico II) and cultured at 37°C and 5% CO_2_ in DMEM medium (Biowest, Nuaillé, France) containing 5% foetal bovine serum (FBS; Biowest). Human melanoma cell lines SAN and A375 were obtained and cultured as previously described [21]. All cell lines used are mycoplasma free. To create FKBP51s overexpressing glioma cells, a True-ORF-Myc-DDK-tagged expression vector was used (OriGene Technologies, Rockville, MD, USA). This vector carried the cDNA of the human FKBP5 transcript variant 4. The relative void vector was also transfected to generate control cells. Transfection was performed using a K2 Transfection System (Biontex, Munich, Germany), according to the manufacturer’s recommendations. For knockdown experiments, cells were transfected with specific short-interfering oligoribonucleotide (siRNA) (or with a nonsilencing oligoribonucleotide [NS RNA] as control) at a final concentration of 50 nM using the K2 Transfection System. NS RNA and siRNA for PD-L1 were purchased from Novus Biological (Littleton, CO, USA). For FKBP51s siRNA, siRNA #1 and #2 were synthesized using target sequences at the 3′-coding region (between 700 and 1100 bp). In addition to siRNA #1 and #2, the siRNA mix contained siRNA #3 (target sequence in the 3′-UTR region, of between 5200 and 5800 bp). Twenty-four hours before transfection, cells were seeded into six-well plates at a concentration of 4 × 10^5^ cells/ml to obtain 80–90% confluence at the time of transfection. Cells were treated 24 h after transfection. A 6 MV X-ray of a linear accelerator (Primus, Siemens, München, Deutschland) was used for the ionizing radiation (IR) experiments. Selective inhibitors of the FK506-binding protein 51 by induced fit (SAFits) were from the laboratory of Prof. Felix Hausch [21]. The anti-PD1 used in the co-cultures was nivolumab (BMS-936558; Bristol-Myers Squibb, Princeton, NJ, USA). For experiments with SAFit compounds (50 μM in DMSO stock solution), 1:1000 DMSO:DMEM-F12 media was used for control cells.

### Immunoblot and immunoprecipitation

Whole cell lysates were homogenized in modified RIPA buffer [15] and assayed by immunoblot. The primary antibody against FKBP51s (rabbit polyclonal of our production, raised against protein C-terminus), CD274/PD-L1 (rabbit polyclonal; Novus Biological) and FKBP51 (rabbit polyclonal; Novus Biologicals) were used diluted 1:2500. For PD-L1 detection, a further rabbit polyclonal antibody Pdcd-1L1 (H-130; Santa Cruz Biotechnology, CA, USA) was used diluted 1:1000. The M2-Flag (mouse monoclonal; Sigma-Aldrich, St. Louis, MO, USA) and γ-tubulin (mouse monoclonal; Sigma-Aldrich) antibodies were used diluted 1:5000. Anti-G3PDH (rabbit monoclonal; Cell Signalling, Danvers, MA, USA) was used at 1:1000. Anti-TGN46 (1:1000; rabbit polyclonal antibody; Sigma-Aldrich); anti-Calnexin (1:1000; AF18, mouse monoclonal; Invitrogen, Carlsbad, CA, USA) and anti-Histone H1 (1:500; AE-4, mouse monoclonal; Santa Cruz Biotechnology) were used as Trans Golgi Network (TGN), endoplasmic reticulum (ER) and nucleus markers, respectively. For co-immunoprecipitation, D54 was used for endogenous pull-down of PD-L1 and FKBP51s. Five-hundred micrograms of total lysate was pre-cleared for 1 h at 4°C, with rotation. Then, 1 μg of each specific antibody was added to the lysate and kept in rotation at 4°C overnight. Twenty microliters of the Protein A-Agarose (Santa Cruz) was added to the mixture and precipitation took place for 2 h, with rotation at 4°C. Samples were then washed in modified RIPA buffer and separated by SDS-PAGE. PD-L1 and FKBP51s antibodies were recognized by immunoblot.

### Immunostaining and microscopy

D54 and U251 were fixed in 4% paraformaldehyde and permeabilized with 0.2% Triton X-100 in 1× PBS for 5 min at room temperature. The non-specific block was performed by treating cells with 10% FBS (Biowest), 1% BSA (Biorad), 0.1% in PBS for 30 min at room temperature. Then, the samples were incubated with primary and secondary antibodies. Rabbit polyclonal, FKBP51s and FKBP51 primary antibodies (see *Immunoblot and immunoprecipitation* section) were used at 1:2000 and 1:50 dilutions, respectively. Alexa Fluor 594 or 488 secondary antibodies were used (1:400; Invitrogen). Nuclei were counterstained with DAPI (Calbiochem-EMD Biosciences, 1:5000). Images were captured with an inverted microscope (DMI4000; Leica Microsystems, Heidelberg, Germany) with Leica Application Suite Advanced Fluorescence (LASAF) software (Leica Microsystems). When required, the brightness, contrast and colour balance of the images were adjusted in Photoshop CS2 (Adobe Systems, San Jose, CA, USA). This adjustment was applied to every pixel in each image.

### Glycosidase digestion

Lysates from D54 cells were subjected to PD-L1 immunoprecipitation. Briefly, 500 μg of total lysate were incubated with 800 ng of anti-PD-L1, or rabbit control IgG (rabbit polyclonal; Santa Cruz Biotechnology), and kept in rotation at 4°C overnight. After 24 h, 20 μl of Protein A-Agarose (Santa Cruz) was added to the mixture and precipitation took place for 2 h, at 4°C in rotation. Samples were then washed thrice in modified RIPA buffer and resuspended in appropriate glycosidase buffer. The N-Glycosidase F (PGNase F) digestion was performed according to the manufacturer’s instructions (CalBiochem Deglycosylation Kit, Merck Millipore, Temecula, CA) to allow N-linked oligosaccharides cleavage of PD-L1, in native conditions. Digested samples were analysed by immunoblot.

### Sub-cellular fractionation

Fractionation was performed according to Sarnataro et al. [28], with little modifications. Briefly, D54 glioblastoma cells were homogenized in 800 μl of Buffer F (0.25 M sucrose, 10 mM HEPES-NaOH, pH 7.2, 10 mM KAc, 1.5 mM MgAc) by pipetting the solution up and down 8–10 times through a 22-gauge needle. The nuclear fraction was sedimented by centrifugation for 5 min at 600×g and then resuspended in RIPA modified buffer. The post-nuclear supernatant was adjusted to 0.75 M sucrose, and the ER fraction was sedimented by ultracentrifugation for 12 min at 16,000 rpm at 4°C, in a Beckman with an SW 50.1 Ti rotor and resuspended in RIPA modified buffer. The supernatant, containing ER fraction, was subjected to immunoprecipitation for PD-L1 as above described. Immunoprecipitated fractions and relative fractionated lysates were analysed by immunoblot (see *Immunoblot and immunoprecipitation* section for antibodies specification).

### qPCR

Serial sections of 10 μm from routinely Formalin-fixed, paraffin-embedded blocks were cut, and total RNA was isolated from eight paraffinized tumours, chosen among those analysed by IHC, by using the High Pure RNA Paraffin Kit (Sigma) according to the manufacturer’s instructions. One microgram of each RNA was used for cDNA synthesis with iScriptTM Reverse Transcription (Bio-Rad, CA, USA). Quantitative gene expression was quantified by qPCR with the 2^−ΔC^_T_ comparative method [29], using the SsoAdvancedTM SYBR Green Supermix (Bio-Rad) and specific qPCR primers to analyse each transcript. To detect FKBP51, validated QuantiTec primers from Qiagen (Valencia, CA, USA) were used. Relative quantitation of the transcript was performed using co-amplified ribosomal 18S [16] and β-Actin [30] as internal controls for normalization.

### Co-cultures of glioblastoma cells and peripheral blood mononuclear cells (PBMCs)

Co-culture was performed as described previously [16]. Briefly, 24 h before starting the co-culture, D54 glioblastoma cells were plated at a concentration of 4 × 10^5^ cells/35 mm plate. After initial plating, the culture medium was aspirated, and adherent cells were washed three times. PBMCs, isolated from heparinized blood by a Ficoll-Hypaque density gradient, were collected and washed by centrifugation. PBMCs were pre-activated with anti-CD3 (eBioscience, San Diego, CA, USA) at the concentration of 5 μg/ml. After 16 h, PBMCs were washed and resuspended in 1% FBS RPMI-1640 medium (Lonza, Belgium) at 1 × 10^6^/ml and 2 ml suspension was added into each glioma cells-precoated plate. Cells were co-cultured at 37°C in a 5% CO_2_ humidified atmosphere in the presence of anti-PD1 (5 μg/ml) or Abciximab (5 μg/ml). For co-culture with SAFit, glioma cells were preincubated with SAFit1 (40 nM) or SAFit2 (60 nM) for 12 h before adding PBMC. For co-culture with PD-L1 silencing, glioma cells were transfected as described in the *Cell culture and transfection* section and, after 24 h, PBMC suspension was added to each glioma cell-precoated plate. After 6 h of co-culture, cells in suspension were collected and separated by centrifugation of supernatants. In each experiment, PBMC was also maintained in mono-cultures as a control. PBMC apoptosis was assayed by flow cytometry.

### Flow cytometry

Expression of PD-L1 was assessed using anti-B7H1-phycoerythrin (PE) (R&D Systems, Minneapolis, MN, USA) at a concentration of 0.05 μg/ml. A relative Ig isotype PE-conjugated antibody was used as a control of non-specific binding. Samples were analysed using a BD Accuri™ C6 Cytometer (BD Biosciences, New Jersey, USA). Analysis of apoptosis was performed by annexin-V staining in double fluorescence with CD45-Peridinin Chlorophyll Protein Complex (PerCP) conjugated. Briefly, 1 × 10^5^ cells were harvested 6 h after co-culture (see co-culture paragraph) and resuspended in 100 μl of binding buffer (10 μM Hepes/NaOH pH 7.5, 140 μM NaCl, and 2.5 μM CaCl_2_) containing 1 μl of annexin-V-FITC (Pharmingen/Becton Dickinson, San Diego, CA, USA) and 5 μl of CD45 for 15 min at room temperature in the dark. Then, 100 μl of the same buffer was added to each sample and analysed with BD Accuri™ C6 Cytometer (BD Biosciences).

### Immunohistochemistry

Tissues were formalin fixed and paraffin embedded. For each case, the most representative block was selected, and two 4 μm thick sections were cut to perform immunohistochemical staining with the anti-FKBP51s primary antibody (see the *Immunoblot and immunoprecipitation* section) at a 1:100 dilution and anti-PD-L1 (Novus; see *Immunoblot and immunoprecipitation* section) at a 1:200 dilution. Sections were dewaxed in xylene, hydrated in a graded series of alcohol and subjected to heat-induced antigen retrieval. After blocking endogenous peroxidase activity, the tissue was incubated with anti-FKBP51s for 60 min and anti-PD-L1 overnight. Subsequently, the slices were rinsed and incubated with the biotinylated secondary antibody at room temperature for 30 min. The bound antibody complexes were stained for 3–5 min, or until appropriate, for microscopic examination with diaminobenzidine, and then counterstained with hematoxylin (30 s) and mounted. Appropriate positive controls for each staining were chosen: normal skin tissue, comprehensive of appendages, for FKBP51s and normal placental tissue for PD-L1. Negative controls were given by sections of glioblastomas treated only with the antibody diluent. The expression of both proteins (FKBP51 and PD-L1) was recorded according to their localization in the cells. Afterwards, a score was established as the product of a proportion score (1: ≤10%; 2: >10% and <50%; 3: ≥50% of tumour cells) and an intensity score (1.weak: the signal was evident only at 40× magnification; 2.moderate: the signal was evident at 20× magnification; **3**.strong: the signal was evident at 10× magnification). The presence of epithelioid tumour cells showing a distinct PD-L1 membranous signal was recorded only if they represented ≥5% of all tumour cells (in accordance with the cut-off used a previous publication) [31].

### Statistical analysis

Student’s t-test was used to analyse differences between means of values. ANOVA served to compare different experimental groups. A p-value of ≤ 0.05 was considered statistically significant.

## SUPPLEMENTARY MATERIALS FIGURES AND TABLES



## Abbreviations

FKBP51: FK506 binding protein 51
FKBP51s: FK506 binding protein 51 short
PD-L1: programmed cell death protein-ligand 1
PD1: programmed cell death protein
PPIase: peptidyl-prolyl isomerase
TPR: tetratricopeptiderepeat
IR: ionizing radiation
ER: endoplasmic reticulum
PNGase F: N-glycosidase F
PBMCs: peripheral blood mononuclear cells.
