# Supplementary material for: A regulatory role for the co-chaperone FKBP51s in PD-L1 expression in glioma
Source: Oncotarget. 2017 Jul 17;8(40):68291–304. doi: 10.18632/oncotarget.19309 (PMC5620257; doi:10.18632/oncotarget.19309)
Supplement: Supplementary file 1 [file oncotarget-08-68291-s001.pdf]

A regulatory role for the co-chaperone FKBP51s in PD-L1 expression in glioma

SUPPLEMENTARY MATERIALS

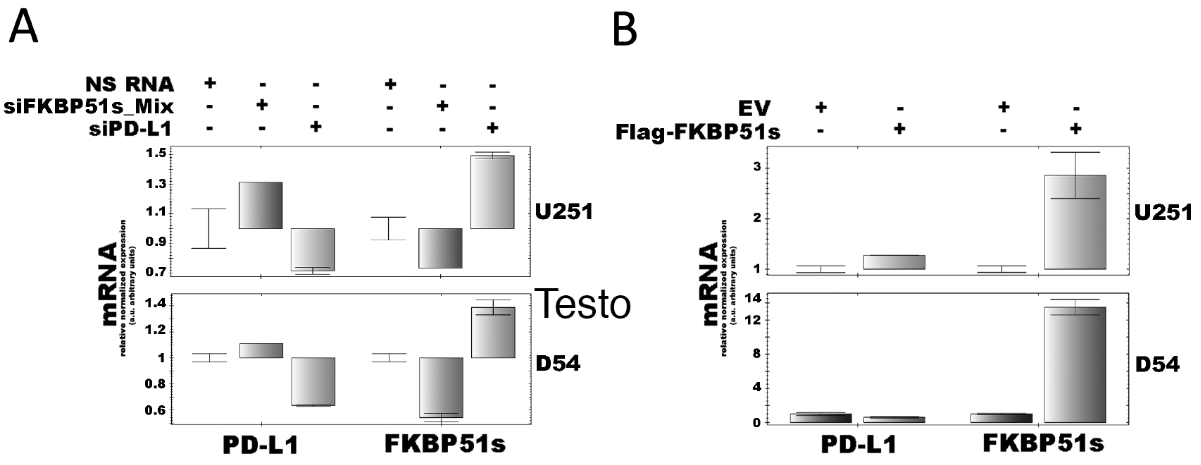

Supplementary Figure 1: Modulation of PD-L1 and FKBP51s transcript levels by gene silencing (A) or over expression (B). (A) QPCR analysis of PDL-1 and FKBP51s mRNA expression levels in U251 (upper) and D54 (bottom) glioma cells, transfected with a NSRNA, FKBP51s siRNA_Mix or a PDL-1 siRNA. **(B)** QPCR analysis of PDL-1 and FKBP51s mRNA expression levels in U251 (upper) and D54 (bottom) glioma cells, transfected with a vector carrying FKBP51s or an empty vector as control.

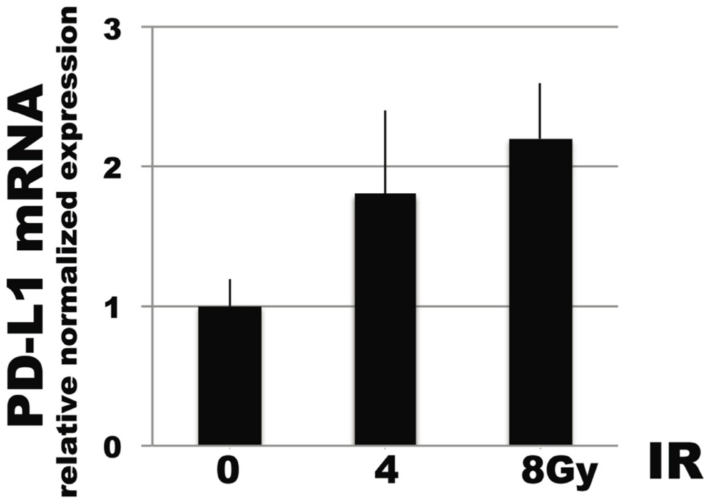

Supplementary Figure 2: IR-induced expression of PD-L1. PDL-1 mRNA expression levels analysis by qPCR in D54 glioma cells, after 4 and 8 Gy irradiation. Cells were harvested 12 h after IR.

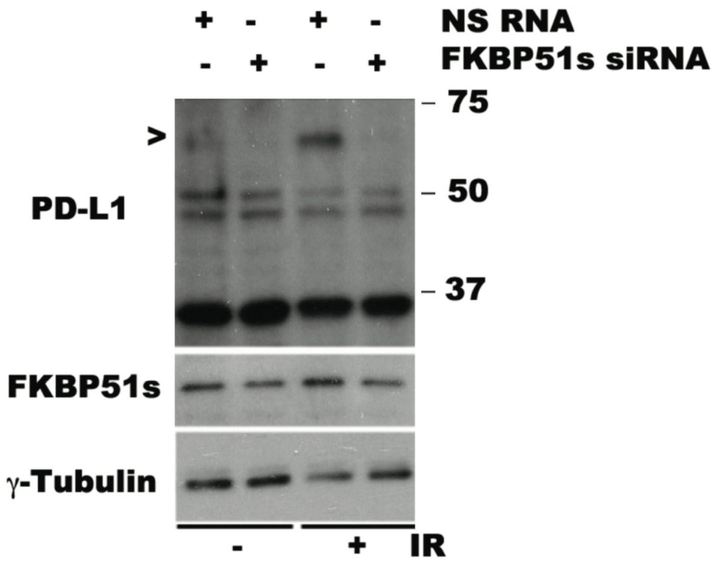

Supplementary Figure 3: IR-induced expression of PD-L1 results impaired by FKBP51s-silencing and Safit. Immunoblot assay of PD-L1 expression in cell lysates obtained from D54 glioblastoma cell, transfected with a FKBP51s siRNAs mix, or a NS RNA, 24 h before a 4 Gy irradiation. Cell was harvested 12 h after IR.

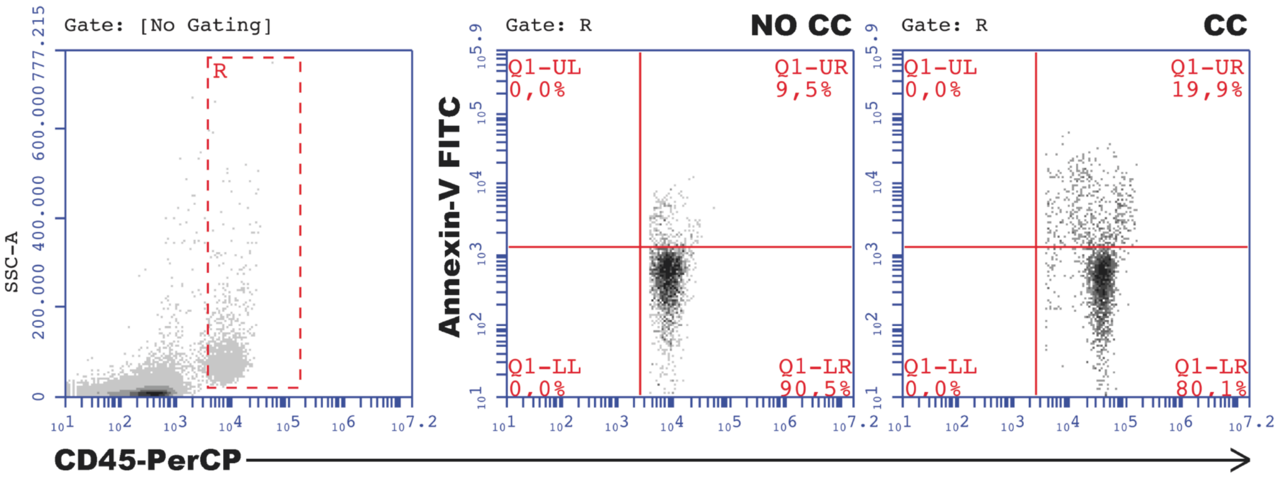

Supplementary Figure 4: CD45-gated PBMCs (left); representative dot plots of the biparametric fluorescence in not co-cultured (NO CC) and co-cultured PBMCs (CC).

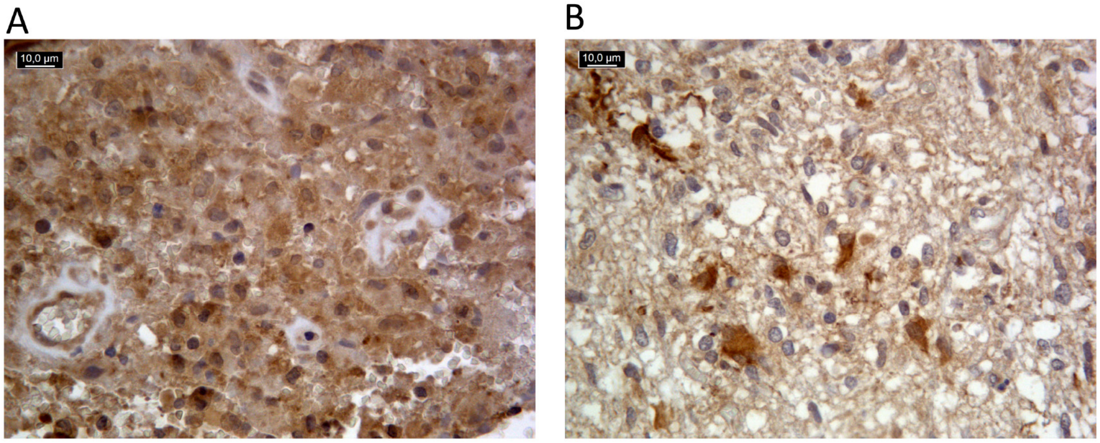

Supplementary Figure 5: PD-L1 immunohistochemistry of glioblastoma samples. **(A, B)** Diffuse/fibrillary cytoplasmic PDL1 expression in glioblastomas: the signal was moderate in case #23 **(A)** and strong in case #27 **(B)**. Original magnification x400.

Supplementary Table 1: Analysis of variance (ANOVA) of cocultured PBMC apoptosis

no CC			CC/D54		
**Treatment**	**Treatment**	**P value**	**Treatment**	**Treatment**	**P value**
None	Abciximab	1.000	None	Abciximab	.839
	Anti-PD1	.418		Anti-PD1	.038
	SAFit1	.418		SAFit1	.065
	SAFit2	.684		SAFit2	.022
Abciximab	None	1.000	Abciximab	None	.839
	Anti-PD1	.418		Anti-PD1	.026
	SAFit1	.418		SAFit1	.044
	SAFit2	.684		SAFit2	.015
Anti-PD1	None	.418	Anti-PD1	None	.038
	Abciximab	.418		Abciximab	.026
	SAFit1	1.000		SAFit1	.787
	SAFit2	.227		SAFit2	.787
SAFit1	None	.418	SAFit1	None	.065
	Abciximab	.418		Abciximab	.044
	Anti-PD1	1.000		Anti-PD1	.787
	SAFit2	.227		SAFit2	.590
SAFit2	None	.684	SAFit2	None	.022
	Abciximab	.684		Abciximab	.015
	Anti-PD1	.227		Anti-PD1	.787
	SAFit1	.227		SAFit1	.590
**CC/U251**			**CC/SF767**		
**Treatment**	**Treatment**	**P value**	**Treatment**	**Treatment**	**P value**
None	Abciximab	.823	None	Abciximab	.212
	Anti-PD1	.003		Anti-PD1	.529
	SAFit1	.001		SAFit1	.089
	SAFit2	.001		SAFit2	.016
Abciximab	None	.823	Abciximab	None	.212
	Anti-PD1	.002		Anti-PD1	.065
	SAFit1	.001		SAFit1	.636
	SAFit2	.000		SAFit2	.212
Anti-PD1	None	.003	Anti-PD1	None	.529
	Abciximab	.002		Abciximab	.065
	SAFit1	.655		SAFit1	.023
	SAFit2	.504		SAFit2	.003
SAFit1	None	.001	SAFit1	None	.089
	Abciximab	.001		Abciximab	.636
	Anti-PD1	.655		Anti-PD1	.023
	SAFit2	.823		SAFit2	.432
SAFit2	None	.001	SAFit2	None	.016
	Abciximab	.000		Abciximab	.212
	Anti-PD1	.504		Anti-PD1	.003
	SAFit1	.823		SAFit1	.432

Supplementary Table 2: Profiles of FKBP51s expression in 29 cases of glioblastoma multiforme

Patient ID	FKBP51s Cytoplasmic	FKBP51s Nuclear	PD-L1 Cytoplasmic	PD-L1 Membranous	OS (months)
	Score *	Score*	Score*	**	
# 1	1	4	4	-	-
# 2	0	4	0	-	3
# 3	1	4	(1x3) 3	-	9
# 4	(3x1) 3	(3x3) 9	(2x3) 6	-	5
# 5	0	(3x2) 6	0	-	12
# 6	0	(1x2) 2	1	-	18
# 7	0	(3x2) 6	(2x3) 6	-	28
# 8	(3x3) 9	(3x1) 3	(3x2) 6	-	23
# 9	0	(2x1) 2	(1x2) 2	-	18
# 10	(3x1) 3	0	0	-	15
# 11	(3x2) 6	(1x2) 2	9	-	15
# 12	(1x3) 3	4	(3x2) 6	-	-
# 13	(2x1) 2	4	4	-	-
# 14	(3x2) 6	9	4	-	-
# 15	4	9	(2x3) 6	-	-
# 16	(3x2) 6	(1x2) 2	9	-	8
# 17	(1x3) 3	(2x1) 2	(3x1) 3	-	10
# 18	0	4	1	-	11
# 19	(3x1) 3	(3x2) 6	4	+	19
# 20	4	(2x3) 6	4	-	18
# 21	1	1	0	-	12
# 22	4	1	9	-	25
# 23	1	(3x2) 6	(3x2) 6	-	29
# 24	1	(2x1) 2	0	-	19
# 25	0	0	0	-	42
# 26	0	(2x1) 2	4	+	38
# 27	0	(2x1) 2	(1x3) 3	-	10
# 28	0	(3x2) 6	(3x1) 3	+	-
# 29	1	1	0	-	24

* (% x intensity)

%(proportion of immunoreactive neoplastic cells): 1, ≤10%; 2, >10% and <50%; 3, ≥50%

Intensity: 1, weak: the signal was evident only at 40x magnification; 2, moderate: the signal was evident at 20x magnification; 3, strong: the signal was evident at 10x magnification.

** +, reaction in ≥5% of tumor cells; -, <5% of tumor cells.
